# Modulation of liver metabolism and gut microbiota by *Alhagi-honey* alleviated heat stress-induced liver damage

**DOI:** 10.1007/s44154-024-00178-6

**Published:** 2024-09-30

**Authors:** Jing Xu, Yundie Liu, Xuanhong Cao, Xinrui Guo, Jie Wang, Yang Liu, Hongda Zhou, Baohua Ma, Sha Peng

**Affiliations:** College of Veterinary Medicine, Northwest A & F University, Yangling, 712100 Shaanxi China

**Keywords:** Alhagi-honey, Gut microbiota, Liver damage, Heat stress

## Abstract

*Alhagi-honey* (AH) is a well-established traditional ethnic medicine with advantageous effects against diarrhea and headaches. We aimed to explore the preventive effect of AH on liver damage induced by heat stress (HS) and its underlying mechanism. HS models were established by thermostat, and mice were treated at 39 ℃ for 10 h, lasting for 7 days. Hematoxylin–eosin (H&E) staining and Periodic Acid-Schiff (PAS) staining were used for histological observation, and transmission electron microscopy (TEM) was used for ultrastructure examination of hepatocytes. Gut microbiota (GM) composition and liver metabolites were respectively analyzed by 16S rRNA sequencing and non-targeted metabolome sequencing. AH pretreatment alleviated liver damage caused by heat stress in mice. The main manifestation was that AH alleviated serum aspartate transferase (AST) and aspartate transaminase (ALT). It was found that AH improved symptoms of hepatocyte damage. In addition, the relative abundance of f_*Rikenellaceae*, g_*Incertae_Sedis* and s_*Staphylococcus_Orisratti*, g_*Lachnoclostridium*, g_*GCA-900066575,* and s_*Alistipes_inops* were modified by AH and these bacterial genera showed association with 6 metabolites (2- (3,4-dihydroxyphenyl) acetamide, 3-hydroxy-3-methylpentanedioic acid, PC (17:0/17:1), Y-L-Glutamy-L-glutamic acid, L-Isoleucine, 5-Methyluridine, 8,8-dimethyl-2-phenyl-4H,8H-pyrano [2, 3-h] chromen-4-one). The Pearson analysis also showed a strong correlation between these microbes and 2 risk indicators (AST and ALT) of liver damage. AH alleviated HS-induced liver damage by regulating liver metabolism and maintaining normal GM. It demonstrated that AH held potential as a prophylactic drug for the prevention of HS-induced liver damage.

**Supplementary Information:**

The online version contains supplementary material available at 10.1007/s44154-024-00178-6.

## Highlights

AH can prevent heat stress-induced liver damage.

AH regulated gut bacterial abundance to promote the production of beneficial metabolites.

AH can prevent heat stress-induced liver damage and its complications as a dietary additive/natural medicine.

**Supplementary Information:**

The online version contains supplementary material available at 10.1007/s44154-024-00178-6.

## Introduction

Every kind of animal has its thermal comfort zone which is crucial to the upkeep of physiological functions, when the temperature exceeds the upper critical temperature of a species (varies by species type), then HS occurs, and the physical workability and motor cognitive ability will be affected by HS, which affects behavior and metabolic materials changes in livestock or even death (Bouchama and Knochel 2002; Rojas-Downing 2017; Ebi et al. 2021).

The liver is one of the most active and important metabolic organs of the human body and plays an important role in the digestion, metabolism, and utilization of nutrients, but it is most susceptible to injury after suffering HS. Numerous studies have shown that the liver metabolism of animals is significantly altered when HS occurs. In the liver, HS affects nucleotide and energy metabolism, amino acid metabolism, glucometabolic, amino acid metabolism, glucometabolic, glycerophospholipid metabolism, linoleic acid metabolism, and sphingolipid metabolism et al. (Fan et al. 2018; Li et al. 2022; Chen et al. 2023). Including the comprehensive effect of systemic krankheit, such as heat cytotoxicity, systemic inflammatory response syndrome coagulopathy, proptosis of hepatocytes, Kupffer cells dysfunction, and abnormally high expression of heat shock proteins, can mediate HS-induced liver damage (Wang et al. 2022). Therefore, it is necessary to take some preventive or treatment measures to reduce HS-induced liver damage.

In addition, GM composition is also impressionable to variable temperatures (Liu et al. 2023). As an important regulator, GM participates in the regulation of the liver by regulating intestinal barrier function, producing metabolites, and mediating immune responses via the gut-liver axis (Tripathi et al. 2018). Normally, GM maintains the integrity of the gut barrier, inhibits the growth of harmful bacteria, and produces metabolites that benefit liver health. However, when intestinal microbes are dysregulated, harmful bacteria will grow and produce harmful metabolites (Tilg et al. 2022; Silveira et al. 2022). Meanwhile, gut permeability increases with increasing temperature, and toxins and pathogens will enter the blood through the gut epithelium, which in turn will contribute to inflammatory immune responses (Blander et al. 2017; Koch et al. 2019; Zong et al. 2020). Studies in recent years also suggested that dysbiosis of GM was involved in the progression of liver damage. Dysbiosis of the GM leads to disruption of the intestinal vascular barrier, which is one of the prerequisites for the development of nonalcoholic steatohepatitis, and it triggers pathological responses in the liver (Mouries et al. 2019; Liu and Yang 2023). Therefore, it is highly likely that HS-induced GM dysbiosis is one of the pro-developmental factors of liver damage. Although in the treatment of acute liver failure and acute hepatic injury secondary to HS, cooling protocols and supportive care are still widespread applications (Davis et al. 2017). However, current research is still insufficient in effectively controlling liver damage caused by HS. Our previous study found that restoring the GM composition was beneficial to help fight HS (Cai et al. 2023). This suggested that maintaining normal GM composition may be a potential strategy to prevent HS-induced liver damage.

Traditional Chinese Ethnic Medicines and their natural products have proved a promise therapeutics for various diseases in recent years, such as Pien Tze Huang, small black bean polysaccharide, and Sheng Mai San which have been proven to suppress Colorectal Tumorigenesis, type II diabetes, and HS-induced liver damage by restoring gut microbiota and producing metabolites (Zhang et al. 2022; Bai et al. 2023; Gou et al. 2023). Alhagi⁃honey (AH) is a traditional Chinese medicine in Xinjiang. Chinese ancient book of Supplement to the Herbal mentions that AH comes from Alhagi. AH is commonly used for relieving abdominal pain, diarrhea, and headaches in Traditional Chinese Ethnic Medicine (Wei et al. 2021). Previous studies have shown that the major component of AH is a polysaccharide that has anti-inflammatory, anti-oxidative properties, protects the intestinal barrier, and treats alcoholic liver disease with effects in modulating the GM. (Wusiman et al. 2022; Song et al. 2024). Our previous studies have found that Alhagi alleviates Ulcerative colitis-induced imbalance of GM composition by exerting the effects of anti-inflammatory and anti-oxidative (Cao et al. 2024). However, there are few studies on AH so far, and it remains unknown whether it could affect the GM of mice and play a role in relieving HS-induced liver damage.

This study aimed to further explore the alleviating effect of AH on HS-induced liver damage and its underlying mechanism. We would use 16S rRNA and non-targeted metabolomics sequencing technology to screen and analyze the differential flora and differential metabolites that may play a beneficial role under the action of AH. Pearson correlation analysis was used to further explore the potential associations and interactions between these differential flora and differential metabolites. This study not only helps to reveal the specific mechanism of AH in protecting heat stress-induced liver damage but also provides a scientific basis for developing new drugs to alleviate HS-induced liver damage and promote the development of new ideas and regimens in related fields.

## Results

### AH alleviated HS-induced liver damage in mice

**Fig. 1:**
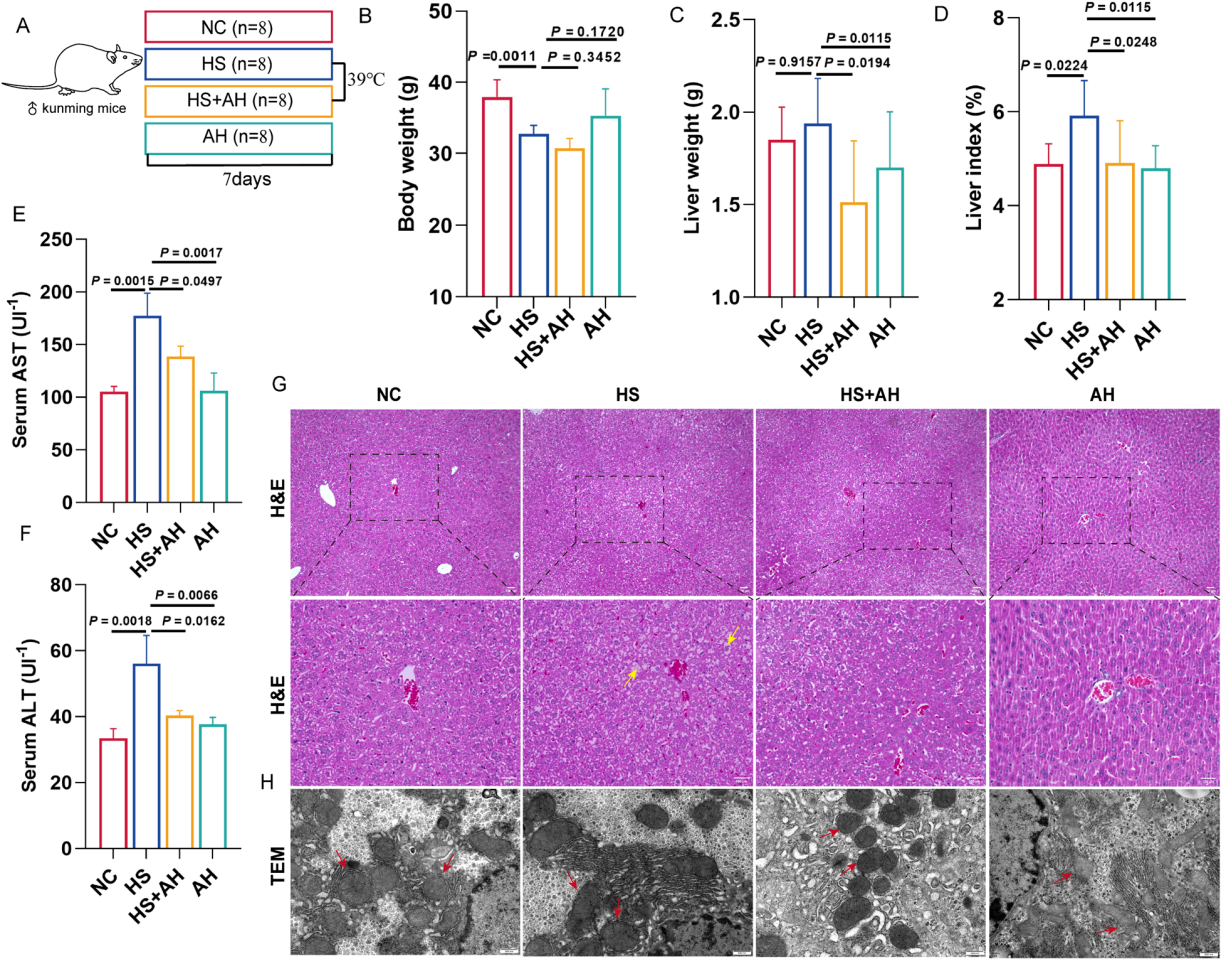
AH alleviated HS-induced liver damage in mice. **A**, Study design of the 39 ℃-induced HS model. **B-D**, Body weight, liver weight, and liver index. 8 mice were used in each group: NC (control with gavage normal saline), HS (heat stress with gavage normal saline), HS + AH (heat stress with gavage *Alhagi-honey*), and AH (control with gavage *Alhagi-honey*). **E–F**, Serum ALT, and AST were detected by a Biochemical analyzer (*n* = 3). **G**, hematoxylin, and eosin (H&E) staining of the mouse liver tissue in 4 different groups. Yellow arrows represent hepatocyte vacuolation and nuclear shrinkage. Scale bar, 100 µm. **H**, representative images of the ultrastructure of liver cells. Red arrows represent mitochondria. Scale bar: 500 nm. All data are presented as the mean of biological replicates ± s.d. One-way analysis and Tukey’s comparisons test were used for *P*-values

To investigate AH’s role in HS-induced liver damage, we established the HS model in mice. Given the strong disease resistance and adaptability of Kunming mice, we chose them to explore the question. Mice in the HS and HS + AH groups were treated at 39 ℃ for 10 h/ day, lasting for 7 days (Fig. 1A). Compared with the NC group, body weights were reduced significantly meanwhile the liver index increased remarkably after HS treatment (*P* = 0.0011 and *P* = 0.0224) (Fig. 1B-D). However, AH treatment reversed the liver and liver index increment induced by HS (*P* = 0.0194 and *P* = 0.0248) (Fig. 1C-D), and this suggested that AH possibly alleviated HS-induced liver damage. Therefore, we detected liver damage marker AST and ALT in serum, and the results showed that AST and ALT were significantly incremented (*P* = 0.0015 and *P* = 0.0018) (Fig. 1E-F), which indicated that the liver damage may occur after HS treatment, but AH pretreatment significantly prevented the serum ALT and AST increasing which were induced by HS (*P* = 0.0497 and *P* = 0.01). Consistent with this result, histological evaluation showed that AH ameliorated HS-induced tissue structure damage, the boundary of hepatic lobule blurred, hepatocyte vacuolation, nuclear shrinkage, irregular arrangement of hepatic cord, and hepatic sinus gap enlargement (Fig. 1G). In addition, the ultrastructure of the cells was used to observe by TEM, and it showed that the morphology, size, and number of liver cell mitochondria were similar in all groups (Fig. 1H). There was no significant difference in mitochondrial morphology and swelling of hepatocytes between the NC group and HS group (Fig. 1H). Together, AH ameliorated HS-induced liver damage in mice.

### AH alleviated HS-induced liver oxidative stress and glycogen synthesis disorders

**Fig. 2:**
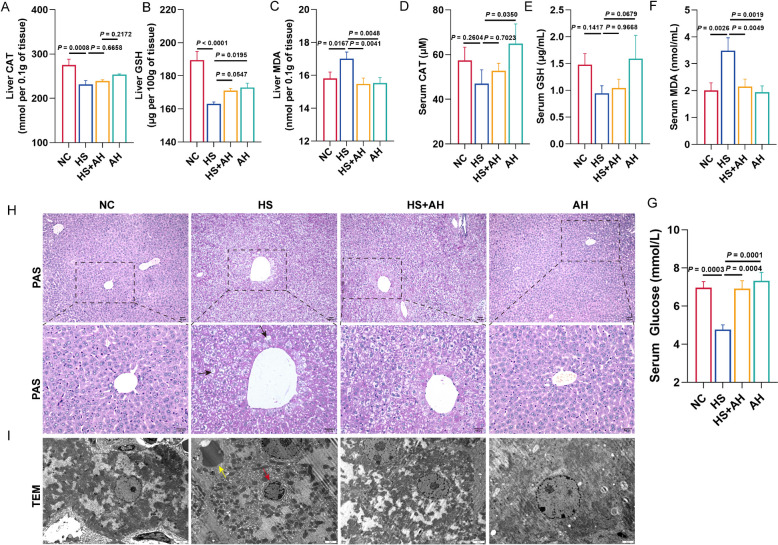
AH alleviated HS-induced liver oxidative stress and glycogen synthesis disorders. **A-C**, Hepatic CAT, GSH, and hepatic MDA. **D**-**F**, Serum CAT, GSH and MDA. (All data were determined according to kit instructions) (*n* = 3). G, Serum glycogen was detected by a Biochemical analyzer. H, Periodic Acid-Schiff staining of the liver from mice treated NC, HS, HS + AH, and AH. The black arrow represents hepatic glycogen breakdown. Scale bar, 100 µm. I, the representative transmission electron microscopy of hepatocytes, the Red arrow represents nuclear shrinkage, and the yellow arrow hepatic glycogenolysis. Scale bar: 2 μm. All data are presented by the mean of biological replicates ± s.d. One-way analysis and Tukey’s comparisons test were used for *P*-values

Liver damage is usually accompanied by the occurrence of oxidative stress. Therefore, the Liver and Serum Catalase (CAT), Glutathione (GSH), and Malondialdehyde (MDA) content were detected by us (Fig. 2A-F). Compared with the NC group, it showed that the Liver and Serum MDA were increased after HS treatment (*P* = 0.0167 and *P* = 0.0026), but AH remarkably decreased the Liver and Serum MDA (*P* = 0.0041 and *P* = 0.0049) (Fig. 2C and F). Compared with the HS group, after HS treatment, AH treatment mitigated the decrease of CAT and GSH to some extent in the liver and serum (Fig. 2A-B and D-E). Previous studies have shown that liver damage could induce glycogen synthesis disorders, therefore we detected the glucose content in serum. The result indicated that serum glucose was decreased after HS treatment (*P* = 0.0003), but AH could inhibit this reduction (*P* = 0.0004) (Fig. 2G). In addition, the histological evaluation showed that AH ameliorated cytoplasmic vacuolation and the reduction of red-colored polysaccharides induced by HS (Fig. 2H). As Fig. 2H shows, we can see the hepatic glycogen breakdown in the HS group was more than in the NC group, and this phenomenon was reversed by AH. This was further verified with transmission electron microscope detection. Compared with other groups, The liver glycogen breakdown and liver cell shrinkage can be seen in Fig. 2I.

### AH regulated the liver metabolic expression profile

**Fig. 3:**
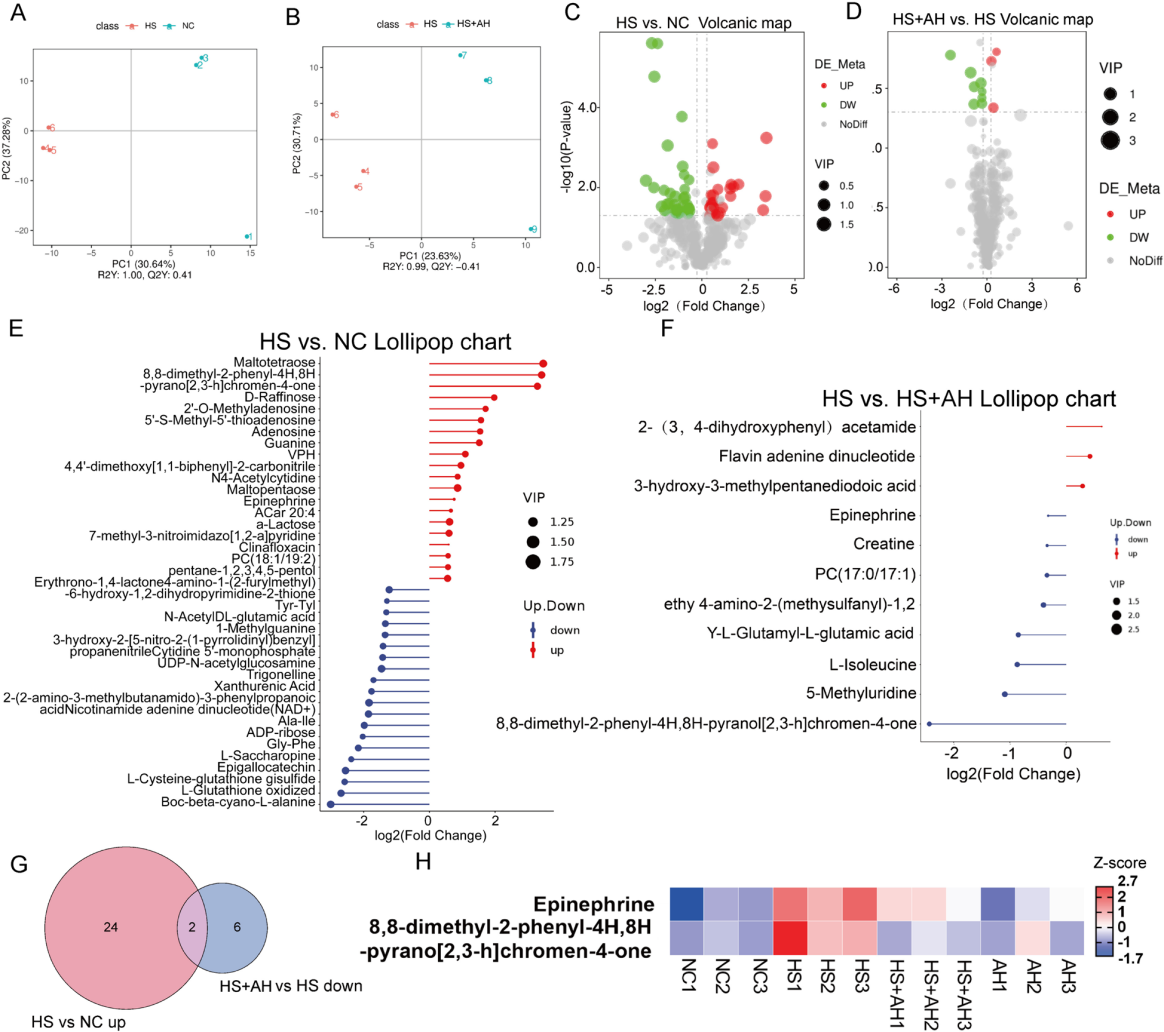
AH regulated liver metabolic expression profile. **A-B**, Partial least squares discriminant analysis (PLS-DA) showing the location of samples(NC and HS) and (HS and HS + AH)from the liver (*n* = 3). **C-D**, the volcano plots show the differential metabolites in the liver (HS and NC) and (HS + AH and HS) (*n* = 3). **E**, the HS-induced top 20 upregulated and down metabolites lollipop chart (*n* = 3). **F**, the AH treatment under HS upregulated and down metabolites lollipop chart (*n* = 3). **G**, Venn of the HS-induced enrichment metabolites and reduced by AH (*n* = 3). **H**, Heatmap of AH-induced changed representative liver metabolites in mice treatment by NC, HS, HS + AH, and AH. Up-regulation or reduction of metabolites induced by different treatments. The color of the diamonds indicates the degree of expression (decreasing from red to blue) (*n* = 3 per group)

To further systemically evaluate the influence of AH on liver metabolism, we conducted non-targeted metabolome profiling in liver samples of the NC, HS, HS + AH, and AH groups. This enabled us to identify 415 metabolites (Data S1), 60 of which were differentially enriched between the NC and HS group, and 11 of which were differentially enriched between the HS and HS + AH group. Partial least squares discriminant analysis (PLS-DA) and Principal component analysis (PCA) (Fig S1A) showed that metabolites in the liver tissues differed between NC and HS, HS and HS + AH groups (Fig. 3A and B). Volcano plots revealed that in the comparison (HS compared to NC), 34 metabolites were down and 26 metabolites were upregulated in the liver (Fig. 3C). We chose the top 20 upregulated and down metabolites to map matches (Fig. 3E). Volcano plots also revealed that in the comparison (HS + AH compared to HS) 8 metabolites were down and 3 metabolites were upregulated in the liver (Fig. 3D). The metabolites of AH-induced significant changes are shown in the lollipop (Fig. 3F). Compared with the HS group, 3 metabolites were enriched by AH, which were 2- (3,4-dihydroxyphenyl) acetamide, Flavin adenine dinucleotide, and 3-hydroxy-3-methylpentanedioic acid (Meglutol) (Fig. 3F). The 2 of 26(HS compared to NC) upregulated metabolites in HS group were down-regulated by AH, which were epinephrine and 8,8-dimethyl-2-phenyl-4H,8H-pyrano [2, 3-h] chromen-4-one (Fig. 3G). We made a heatmap of the relative expression of these metabolites, and it clearly showed that the enriched 2 metabolites in the HS group were reduced by AH (Fig. 3H). Surprisingly, AH can also enrich beneficial metabolites in the liver without HS treatment, such as 5-hydroxytryptophan, L-Phenylalanine, and Ornithine (Fig S1B).

In the analysis of metabolite KEGG enrichment (Fig S1C), we found that AH enriched a large number of protein processing-related metabolic pathways. Therefore, serum levels of total protein (TB), albumin (ALB), serum globulin (GLO), and ALB/GLO were measured. The results showed that the trend of HS-induced protein increase was further increased by AH (Fig S2A-D). The slightly decreased albumin in the HS group was upregulated by AH (Fig S1B). The increase of the A/G HS group was significantly decreased by AH (Fig S2D). Because both metabolomic and serological evidence demonstrate that AH promotes protein processing, TEM was used to observe the ultrastructure of cells. The images showed that AH significantly increased protein processing (Fig S2F). The endoplasmic reticulum area was analyzed with Management Software Computer Aided Design (CAD) modeling and the ribosome was counted with image-J, and then the ribosome binding rate in the same area was compared. The results were consistent with the metabolomics sequencing results. AH, significantly increased protein synthesis (Fig S2E). Taken together, these results reveal that the liver could produce beneficial metabolites after AH treatment, and these metabolites may ameliorate HS-induced liver damage to some extent.

### AH regulated the composition of gut microbiota

**Fig. 4:**
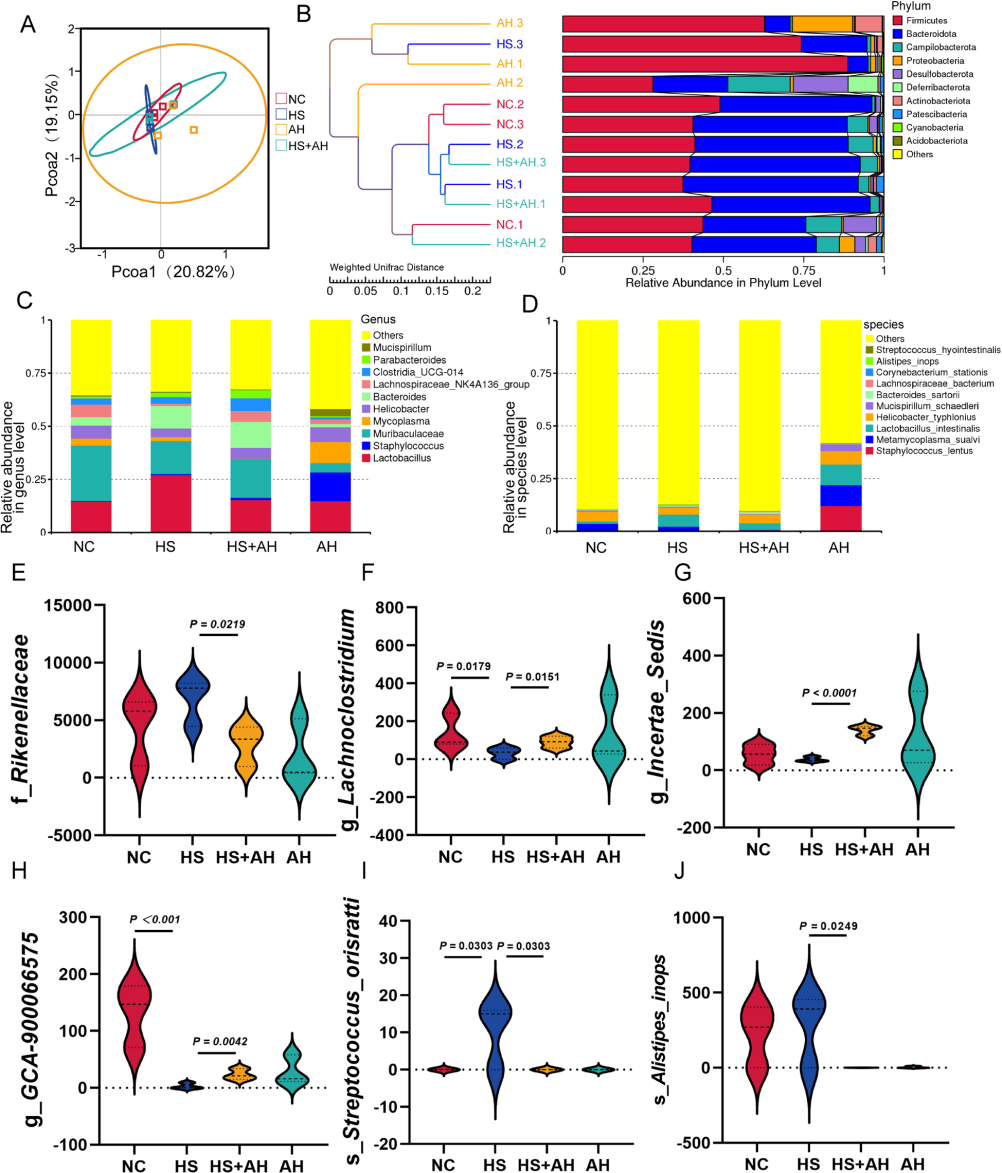
AH regulated the Structure of gut microbiota. **A** PCoA analysis (beta diversity) of the gut microbiota in each group (*n* = 3). **B**, Relative Abundance in Phylum level of each group (*n* = 3). **C**, Relative Abundance in Genus level of each group (*n* = 3). **D**, Different levels of microflora between NC group and HS group **E**, The main reduced flora by HS in genus level (*n* = 3). **F**, The main enrichment flora by AH in genus level. (*n* = 3 per group). All data are presented by the mean of biological replicates ± s.d. One-way analysis of variance was used for *P*-values

Given that the Structure of gut microbiota is closely associated with liver metabolism in the previous study. To explore the potential mechanism for the mitigative effect of AH in liver damage, liver oxidative stress glycogen synthesis disorders, and metabolic disorders, we studied the composition changes in gut microbiota in response to AH by 16S rDNA sequencing technology. As shown in Fig S3A, α-diversity indexes(Chao 1, observed features and dominance) of the gut microbiota in HS + AH were a little bit higher than that of HS, and no significant differences in the α-diversity were discovered among all groups. But the Bray Curtis distance (PCoA) analysis models also showed AH has changed the intestinal flora of HS to some extent (Fig. 4A). According to the Unweighted Pair-group Method with Arithmetic Mean (UPGMA) tree, we found AH and NC were far apart, which indicated that gut microbiotas were changed by AH (Fig. 4B). There are mainly Bacteroidetes, Firmicutes, and Campilobactrota in fecal microbiota at the phylum level (Fig. 4B). It’s Lactobacillus, Staphylococcus, and Muribaculaceae that dominate at the genus level (Fig. 4C). At the species level, intestinal dominant bacteria are mainly s_*Staphylococcus_lentus*, s_*Metamycolasma_sualvi,* and s_*Lactobacillus_intestinalis* (Fig. 4D). From these figures, it could be seen that AH mainly recovered the flora at the genus level (Fig. 4B-D). Therefore, we mainly focus on the microflora differences at the genus level.

To identify the flora that differed significantly between the groups, The hypothesis testing of inter-species abundance was Metastat to obtain *P*-values, and species with significant differences between groups were screened according to *P*-values. Different from the HS group, AH treatment remarkably reduced the abundance of f_*Rikenellaceae,* g_*Incertae_Sedis,* and s_*Staphylococcus_Orisratti* (Fig. 4E, G, and J). Beyond that, compared with the NC group, the abundance of g_*Lachnoclostridium,* g*_GCA-900066575,* and s_*Alistipes_inops* were significantly reduced after HS treatment, but AH treatment could conspicuously mitigate this trend. In addition to this, we found that the intestinal microbiota of mice treated only with AH also had significant changes (Fig. 4B-D). Our data showed that AH restored the intestinal flora disturbance induced by HS to a certain extent, especially at the genus level.

### Gut microbiota was involved in regulating liver metabolism

**Fig. 5:**
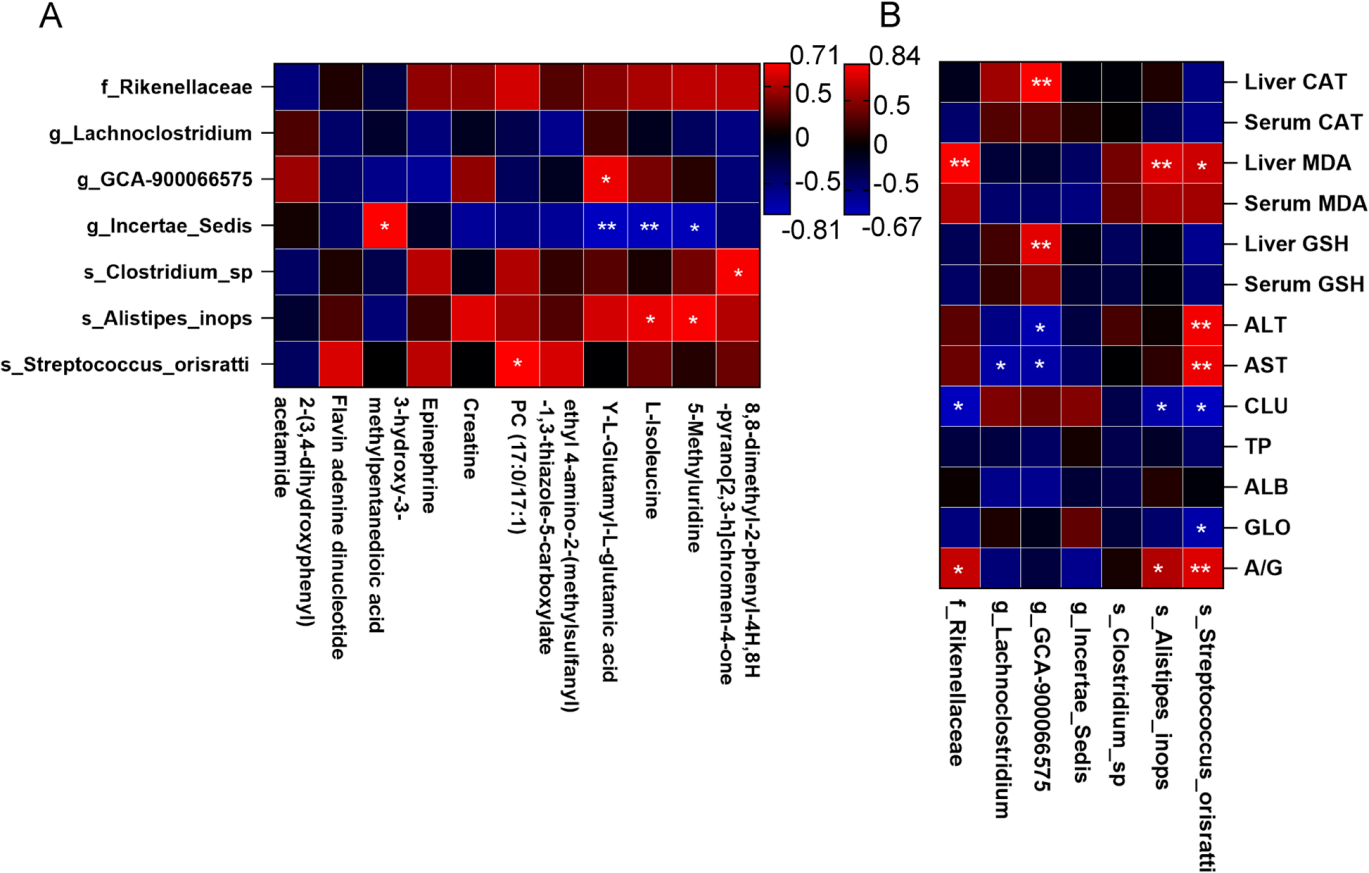
Gut microbiota was involved in regulating liver metabolism. **A**, Pearson correlation analysis of metabolites in the liver and the abundance of main gut microbiota. **B**, Pearson correlation analysis of inflammatory parameters, anti-oxidative, or liver damage makers in Serum and the abundance of main gut microbiota. The red color denotes a positive correlation, while the blue color denotes a negative correlation. The intensity of the color is proportional to the strength of the Spearman correlation. *P < 0.05, **P < 0.01

Gut microbes influence liver metabolism through enterohepatic axis mediation (Tripathi et al. 2018), we explored the potential relationship between gut microbiota and liver metabolism by Pearson correlation analysis, and in Fig. 5A and B, we respectively used correlation coefficient ranges of (-0.81, 0.71) and (-0.67, 0.84). The relationship between the AH-induced major gut microbiota and the AH-induced liver metabolites was analyzed. We found that g_*Incertae_Sedis* was significantly negatively correlated with many of the metabolites reduced by AH, such as Y-L-Glutamy-L-glutamic acid (R = -0.817, *P* < 0.01), L-Isoleucine(R = -0.802, *P* < 0.01) and 5-Methyluridine(R = -0.791, *P* < 0.05), and was significantly positively correlated with 3-hydroxy-3-methylpentanedioic acid(R = 0.733, *P* < 0.05). G_ *Clostridium_sp* was significantly positively correlated with 8,8-dimethyl-2-phenyl-4H,8H-pyrano [2, 3-h] chromen-4-one(R = 0.714, *P* < 0.05), and s_*Alistipes_inops* was significantly positively correlated with L-Isoleucine(R = 0.672, *P* < 0.05) and 5-Methyluridine. S_*Streptococcus_orisratti* (R = 0.723, *P* < 0.05)was significantly positively correlated with PC (17:0/17:1) (R = 0.735, *P* < 0.05). We also explored the correlation between gut microbes and serum biochemical, oxidative stress markers, and liver damage makers. The results indicated that f _*Rikenellaceae*(R = 0.839, *P* < 0.01), s_*Alistipes_inops*(R = 0.727, *P* < 0.01) and s_*Streptococcus_orisratti*(R = 0.669, *P* < 0.05) was obviously positively correlated with liver MDA. Probiotics g_*Lachnoclostridium*(R = -0.583, *P* < 0.05) and g_*GCA-900066575*(R = - 0.586, *P* < 0.05) was negatively correlated with AST. These results indicated the interaction between gut microbiota and liver metabolites took part in the modulation of serum glucose homeostasis and indexes in mice.

## Discussion

Chinese Traditional Ethnic Medicine is a promising strategy for alleviating HS-induced liver damage. In this study, we explored the protective effect of Chinese Traditional Ethnic Medicine AH in liver damage induced by HS in mice. The manifestations of liver damage were mitigated by AH administration, including decreased liver index, serum liver damage markers, remission of glycogen synthesis disorders, maladjustment of liver metabolic expression profile, and GM composition. We found that the important microbiota showed a significant association with 6 AH-induced liver metabolites and the liver damage makers (serum AST and ALT) through Pearson correlation analyses. These results suggested that AH is a promising Chinese Traditional Ethnic Medicine for acute hepatic damage prevention in the context of HS.

We noticed that the HS-induced liver enlargement, degeneration, and necrosis of liver tissue structure (Fig. 1G). Meanwhile, we also noted an increase in AST and ALT in the blood. The elevation of AST in the blood suggested the possibility of liver cell or mitochondrial damage in our liver. However, relying on TEM, we found that mitochondria in hepatocytes were not damaged (Fig. 1H). AST is widely present in a variety of tissues and cells in the whole body, mainly in the heart and liver, and AST is in the mitochondria of hepatocytes under normal physiological conditions (Sookoian and Pirola 2015). AST in the liver has two isoenzymes, mAST in the mitochondria and sAST in the cytosol. sAST is released into the blood during mild hepatocyte lesions. We speculated that the increase in serum ALT and AST may come from the outflow of ALT and AST from the cytoplasm into the blood after cell membrane injury. This may be because we used a chronic heat stress model, resulting in liver damage caused by heat stress being limited to the cell membrane. It has also shown that HS can damage the integrity of cell membranes (Emami et al. 2020), which is also consistent with our conjecture.

What’s more, we also found that AH can ameliorate HS-induced hepatic oxidative stress and glycogen synthesis disorders to a certain extent. Compared with the NC group, serum glucose decreased significantly in the HS group, and AH significantly improved the serum glucose levels (Fig. 2G), but this was not consistent with histological and cytological evidence. We could see that liver glycogen breakdown decomposition under HS was more than that in the NC (Fig. 2H). Based on TEM, there were a lot of lipid droplets and glycogen in liver cells of the HS group (Fig. 2I). This may be a compensatory phenomenon of decreased appetite and accelerated metabolism induced by HS, resulting in decreased blood glucose in the body and accelerated glycogen breakdown in the liver. In HS broiler models, a similar phenomenon was found. Liver oxidative damage, liver glycogen infiltration, and hepatic lipid accumulation were found in the liver of broilers when HS occurred, with mitochondrial tricarboxylic acid (TCA) cycle abnormalities and mitochondrial dysfunction (Jing et al. 2023).

Based on these phenomena, we found that AH has a better protective effect on the liver during HS. The metabolic profile of the liver was analyzed. It’s easy to see from Fig. 3B that AH pretreatment significantly changed the metabolic profile of the liver and the location of HS + AH was between NC and HS. In other words, AH had a salvage effect on HS-induced metabolic disorders. Given that AH was protective against liver damage, we inferred that the protective effect of AH might be attributed to modulating the liver metabolome to protect hepatocytes from HS damage. Relying on liver metabolomics sequencing, we found several key metabolites, including epinephrine and 8,8-dimethyl-2-phenyl-4H,8H-pyrano[2,3-h] chromen-4-one. They probably were the metabolites that AH works on. In agreement with our results, a rise of epinephrine in the liver has been shown to cause liver glycogen breakdown in vivo (Ottolenghi et al. 1985). Beyond that, epinephrine was proven to enhance the proliferation of intrahepatic cholangiocarcinoma (iCCA) cells in vitro (He et al. 2023). At the same time, AH also upregulates beneficial metabolites in the liver, including Flavin adenine dinucleotide and 3-hydroxy-3-methylpentanedioic acid (Meglutol). It’s worth mentioning that Flavin adenine dinucleotide was permitted for use to treat vitamin B2 deficiency in Japan under the trade name Adeflavin. Meglutol is a widely used drug, which was verified to decrease plasma ketone bodies, triglycerides, and slight total cholesterol (Francesconi et al. 1987). In addition, we also found an interesting phenomenon, that AH significantly increased the ribosome and ER binding rate. We hypothesized that AH increases protein synthesis in the liver. This indicated that AH could alleviate HS-induced liver damage by enhancing the expression of beneficial metabolites and reducing the expression of harmful metabolites.

Large-scale epidemiological research has shown that gut microbiota potentially impacts the metabolism of the host (Sheflin et al. 2017; Pedersen et al. 2016). The health of the gut is linked to the health of the liver. We next elucidated the events by which AH regulated the microbiome. We found that the abundance of some gut-beneficial flora decreased significantly after HS treatment. For genus level, some gut probiotics were reduced due to HS-treated. Of these, g_*Lachnoclostridium* is the bacteria that has been shown to produce (short chain fatty acid, SCFA) (Yao et al. 2020). G_*Lachnoclostridium* belongs to the family Lachnospiracea, and its anti-inflammatory potential has been reported in previous research (Komiyama et al. 2021; Sehgal et al. 2020). This was consistent with our research that g_*Lachnoclostridium* was negatively correlated with Serum AST. After intervention with AH, the relative abundance of g_*Lachnoclostridium* was recovered. G_*GCA-900066575* has been reported to be enriched after recovery in a variety of gut microbiota disorders (Cai et al. 2023; Guo et al. 2023), and AH pretreatment also significantly increased the abundance of this bacteria in the gut compared with HS groups. The abundance of g_*Incertae_Sedis* was significantly increased by AH pretreatment. However, less research has been studied on g_*Incertae_Sedis*, and given its negative association with harmful metabolites, we hypothesized that it might be a gut-friendly bacterium. Along with these, we found that AH pretreatment also decreased some bacteria. Such as f_*Rikenellaceae*, which is highly enrichment in depressed with gut microbiota-disturbed mice (Duan et al. 2021). Notably, a significant positive correlation between the abundance of s_*Staphylococcus_Orisratti* and liver metabolite PC (17:0/17:1), Serum AST, and ALT concentration was found. These results support our discovery in this research and further indicate that AH restores the gut microbiota disorder HS-induced to a certain extent.

In the present study, we focused on the effects of AH on liver metabolism and GM at the individual level and conducted a preliminary mechanistic exploration. However, the components of AH are also relatively complex, and we did not explore which molecule was responsible. In addition, the target organs, target cells, and organelles of AH work are also unknown. To clarify these, we would need to perform subcellular analyses, but at present AH is a mixture. We need to further explore the substances by which AH exerts the protective effects described here, as well as pharmacokinetic studies in major organs. After identifying the molecules of AH, we can further investigate the mechanisms of interaction between molecules cells, and organelles. These are our next research directions.

**Fig. 6:**
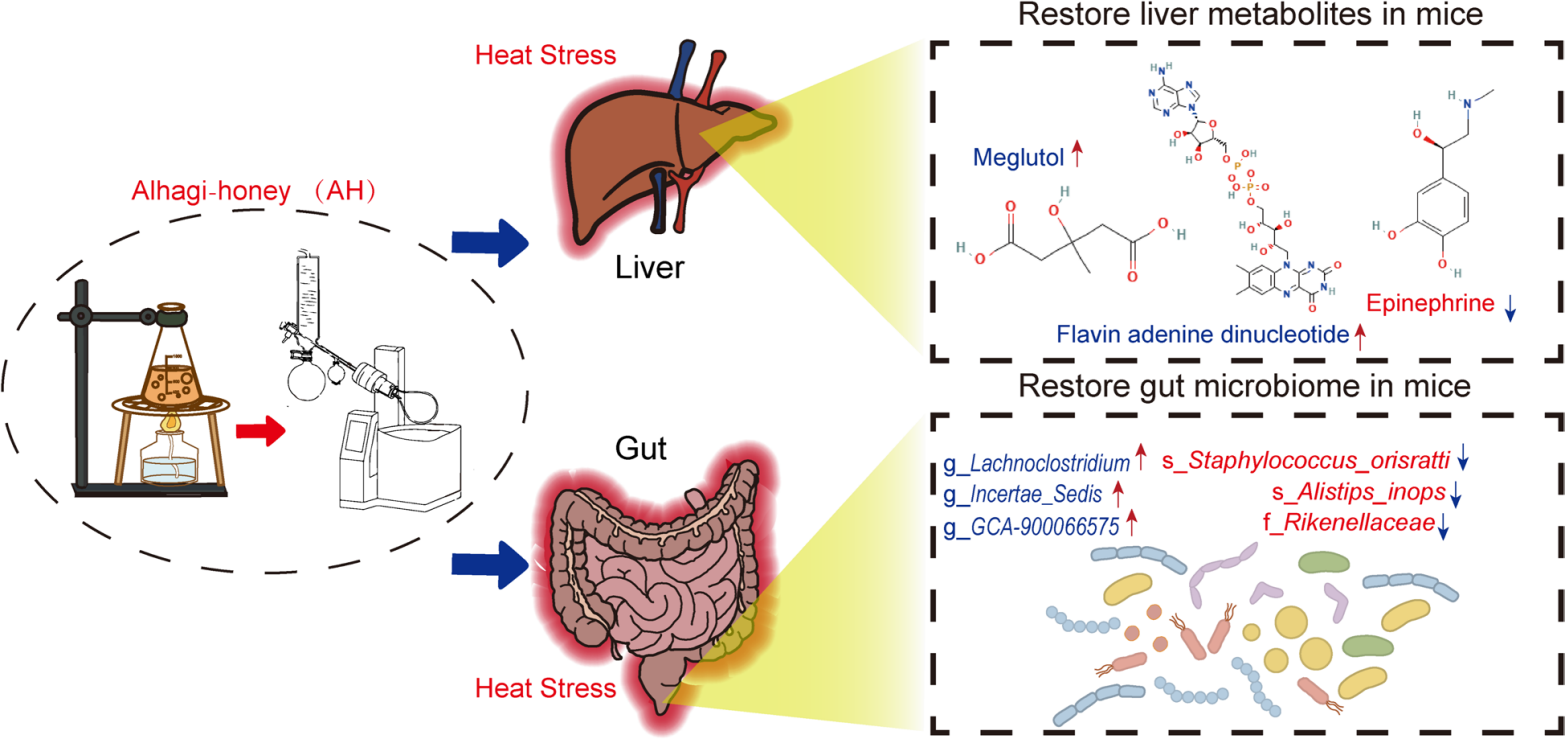
AH influenced liver metabolites and gut microbiota. Prefeeding AH before HS alleviated body weight ratios, serum AST and ALT gain in HS exposure by elevating beneficial metabolite Meglutol, Flavin adenine dinucleotide and reducing harmful metabolite epinephrine in the liver, as well as increasing gut beneficial microbiota(g_*Lachnoclostridium*, g_*Incertae_Sedis* and g_*GCA-900066575*) and decreasing harmful(s_*Staphylococcu, s_ Orisratti*, s_*Alistipes_inops* and f_*Rikenellaceae*) microbiota

To sum up, we discovered the novel effect of AH in addition to the traditional treatment of diarrhea and headache, which AH alleviated HS-induced liver damage by regulating liver metabolism and gut microbiota (Fig. 6). Thus, AH is a potential drug to prevent of liver damage induced by HS in mice.

## Materials and methods

### Ethics statement

Male Kunming mice (30 ± 5 g,  8 weeks old) were used in this experiment, and we purchased these mice from CHENGDUDOSSY. Our studies were following the Guidelines for the Care and Use of Laboratory Animals and approved by the Animal Use Ethics Committee of Northwest A&F University (Approval No. 201902A299).

### Preparation and quality control of AH

AH was purchased from the Xinjiang Uyghur Ethnic Hospital (Xinjiang, China). AH was extracted by the method of water extraction. Add 100 g AH to 1 L distilled water and boil in a flask for 2 h. Strain and add 500 mL distilled water and bring to a boil. The filtrate is mixed twice and concentrated in a rotary vacuum evaporator. AH extracts were freeze-dried at -80 ℃ and refrigerated at 4 ℃ for later use.

The clinical dosage of AH was 4 g. In this study, a moderate dose of AH was given, and its normal dose corresponded to the human clinical dose. The dose calculation formula of AH is as follows. Dm = Dh/W × F (the working dose of mice is Dm. The clinical dose is Dh. Human body weight is W which is set to 60 kg. F is the dose conversion coefficient between mice and humans which is equal to 12.3). AH-treated mice were intragastric with a corresponding dose of AH before heat exposure 2 h, and it lasted for 7 consecutive days. The dose of medication was also referred to in the previous study (Song et al. 2024; Cao et al. 2024), and united previous calculations, finally, the intermediate dose was selected.

### HS mouse models and drug administration

The heat stress model was developed in our previous publication (Cai et al. 2023). Specifically, normal feeding for 7 days before the experiment. 32 Kunming mice were randomly divided into 4 groups (*n* = 8). Mice in the natural control (NC) group and AH control (AH) group were kept in pathogen-free animal facilities in ventilated cages at 22 ℃, 30 ± 5% humidity. The heat stress (HS group) and heat stress with AH (HS + AH group) of the mice were exposed to 39 ℃, 30 ± 5% humidity incubator from 8. 00 AM to 18. 00 PM at day and recovered at 22 ℃, 30 ± 5% humidity at night. All mice had a 12/12 h diurnal cycle and were fed continuously for 7 days. AH is dissolved in saline, and it is administered at a dose of 125 mg/mL, 0.2 mL/mice. The NC and HS groups were given the same amount of saline.

### Sample collection

After HS treatment for 7 days, mice were killed after isoflurane anesthesia in a fasting state. Blood samples were isolated by centrifugation at 3000 g for 15 min to obtain serum. The liver tissue was removed and stored in 4% paraformaldehyde. The liver histological changes were observed in pathological sections. Liquid nitrogen was used to freeze liver tissue and stool then stored at -80 ℃ for sequencing.

### Detection of serum parameters

The kits of CAT, GSH, and MDA used in this experiment were purchased from Abbkine (Wuhan) Biotechnology Co., LTD. After the collected serum and tissue samples were treated according to the kit instructions, the absorbance of the samples at specific wavelengths was determined using the Thermo Scientific Microplate Reader (Thermo Fisher, American), and the concentration and reliability of biochemical indicators in the samples were calculated according to the formula provided in the kit instructions, and the data of each sample was analyzed and processed (KTB 1040, KTB 1600, KTB1050). The serum biochemical indices were detected by a fully automatic biochemical instrument (BK-400 BIOBASE). The detection indicators were AST, ALT, CLU, TP, ALB, GLO, and A/G.

### Histological analysis

After the liver was removed, it was soaked in 4% paraformaldehyde (PFA) and fixed for 24 h. Graded ethanol was used to dehydrate, embedded in paraffin, and cut into 2 μm slices. The slices were fixed in the Bouin solution for H&E staining and PAS staining.

### TEM (transmission electron microscopy)

The removed liver tissue was first prefixed with 3% glutaraldehyde and then re-fixed with 1% osmium tetroxide. The fixed tissue was dehydrated step by step with acetone, and Ep812 encapsulated the dehydrated tissue. A glass knife machine (EM KMR3, Leica) is used to make semi-thin slices. Optical positioning was achieved by dyeing semi-thin slices with toluidine blue, followed by diamond knives (EM UC7, Leica) to make ultra-thin slices, and finally staining with uranium acetate and lead citrate. The sections were observed using TEM (JEM-1400-FLASH, JEOL).

### Non-targeted metabolomics sequencing

Liver tissue metabolome sequencing was performed by Novogene Co., Ltd. (Beijing, China). Metabolites were extracted from mouse liver using a mixture of methanol and aqueous solution (Want et al. 2013). HypersilGoldcolumn(C18) column (100 × 2.1 mm, 1.9 μm) was used for chromatographic separation at 40 ℃. The 0.1% formic acid and 5 mM ammonium acetate were respectively the mobile phase A of positive and negative ions, and the mobile phase B is methanol. The flow rate was 0.2 mL/min. The scanning range was m/z 100–1500. ESI source Settings were as follows: Spray Voltage: 3.5 kV; Sheath gas flow rate: 35psi; Aux Gas flow rate: 10 L/min; Capillary Temp: 320 °C; S-lens RF level: 60; Aux gas heater temp: 350 °C; Polarity: positive, negative; MS/MS second-level scans were data-dependent scans.

The raw data files from UPHPLC-MS/MS were processed by Compound Discoverer 3.1 (CD3.1, ThermoFisher) to carry out peak picking, peak alignment, and quantification for each metabolite. The main parameters are as follows: Retention time: 0.2 min; Mass deviation: 5 ppm; Signal strength deviation: 30%; Signal to Noise Ratio: 3; Minimum signal strength, et al. Then, the peak was extracted by adding and summing ion peaks, while the peak area was subjected to air volume, the target ions were integrated, and finally, the molecular formula was predicted by molecular ion peaks and fragment ions. MzCloud(https://www.mzcloud.org/), mzVault, and Masslist database were used to compare the disembarkation data. The KEGG((https://www.genome.jp/kegg/pathway.html), HMDB((https://www.genome.jp/kegg/pathway.html), and LIPID(http://www.lipidmaps.org/) database were used to identify the metabolites. The metaX software was used for data conversion and PCA and PLS-DA analysis, and VIP values for each metabolite were obtained. The multiple metabolite difference (FC) and *P*-value between the two groups were calculated using the T-test (Wen et al. 2017). VIP > 1, *P* < 0.05, and FC ≥ 2 or FC ≤ 0.5 were used to screen for differential metabolites as the standard. The R package ggplot2 was used to map volcanoes and bubbles. We used the KEGG database to find out the function and metabolic pathways of metabolites. The relative expression heat map of metabolites was drawn using GraphPad Prism 9.5, and *Z*-score was used to normalize (Haspel et al. 2014; Heischmann et al. 2016).

### 16S rRNA sequencing

Each group of feces was sent to Novogene. Genomic DNA was extracted according to the kit (TianGen) instructions. Bacterial diversity was identified using 16S V3-V4 region primers (515F and 806R). The mixture of PCR was then added with 15μL High-Fidenlity PCR Master Mix (Phusion), 0.2 μM primers, and 10 ng genomic DNA template, denatured at 98 ℃ for 1 min. 30 cycles were performed at 98 (10 ℃), 50 ℃ (30 s), and 72 ℃ (30 s), with the last 72 ℃ for 5 min. PCR products were identified and recovered with a general-purpose DNA purification kit (Tiangen). A library construction kit (TianGen) was used to construct the library. The Qubit and Q-PCR were used to quantify, then the PE 250 computer sequencing was performed using NovaSeq 6000. FLASH (Version 1.2.1), FASTP (Version 0.23.1), and the Species annotation database were used to control data quality to obtain valid data. Species annotation and phylogenetic tree were constructed using QIIME2 (Version QIiME2-202006) and observed_otus and chao1 indices were calculated.

### Metabolome and gut microbiota correlation analysis

Correlations between important metabolites and bacterial populations were analyzed by Pearson correlation coefficient using IBM SPSS Statistics 26.

### Statistical analysis

Except for the specified statistical analysis used in metabolomics and microbiology experiments, univariate ANOVA and unpaired T-tests were performed using GraphPad Prism 9.5 for other data. *P* < 0.05 is considered statistically significant.

## Supplementary Information

Supplementary Material 1.

Supplementary Material 2: Fig S1. AH changed liver metabolic profile. A, PCA analysis of each samples. B, Lollipop chart of AH vs.NC. C, KEGG enrichment analysis of AH. All *P* < 0.05.

Supplementary Material 3: Fig S2. AH promoted protein processing. A-D, Serum ALB, GLO and A/G were detected by a Biochemical analyzer. E, Representative images of the ultrastructure of liver cells. Scale bar, 500 nm. F, Endoplasmic reticulum-ribosome(X/1000 nm^2^) was counted by Image-J and CAD. Data are presented by the mean of biological replicates ± s.d. One-way analysis of variance was used for *P*-values.

Supplementary Material 4: Fig S3. The α-diversity including Chao1, observed features and dominance. A, chao1 index of each treatment group. B, observed_teatures index of each treatment group.

## Data Availability

All data generated or analyzed during this study are included in this published article and its supplementary information files. The datasets supporting the results and conclusions of this article were deposited in the NCBI Sequence Read Archive database under the accession number PRJNA1103337 (microbiota sequencing data) and the data on metabolomics were deposited in the https://www.ebi.ac.uk/metabolights under the accession number MTBLS10021 with URL www.ebi.ac.uk/metabolights/MTBLS1002 (Yurekten et al. 2024). All other data are contained within the main manuscript.
