# Supplementary figures and images for: Modulation of liver metabolism and gut microbiota by Alhagi-honey alleviated heat stress-induced liver damage
Source: Stress Biol. 2024 Sep 30;4(1):41. doi: 10.1007/s44154-024-00178-6 (PMC11442815; doi:10.1007/s44154-024-00178-6)

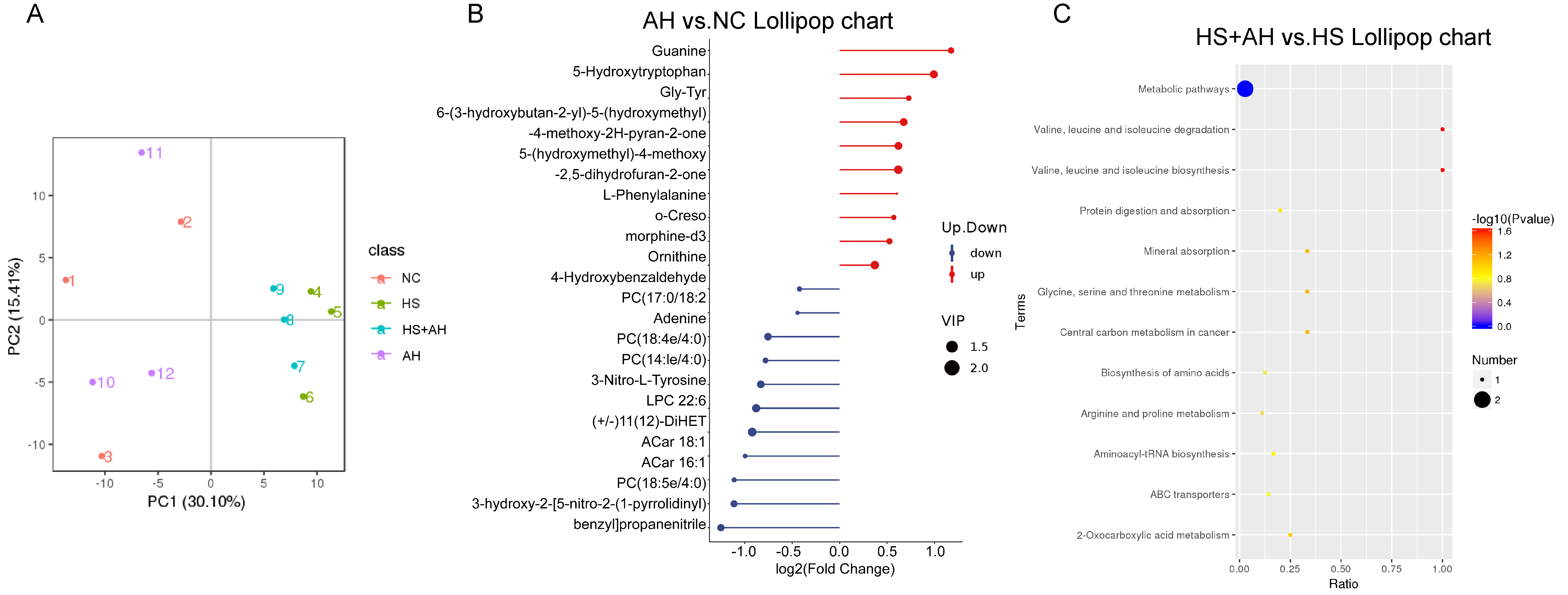

Supplement: Supplementary file 2 — Supplementary Material 2: Fig S1. AH changed liver metabolic profile. A, PCA analysis of each samples. B, Lollipop chart of AH vs.NC. C, KEGG enrichment analysis of AH. All P < 0.05. [file 44154_2024_178_MOESM2_ESM.tif]

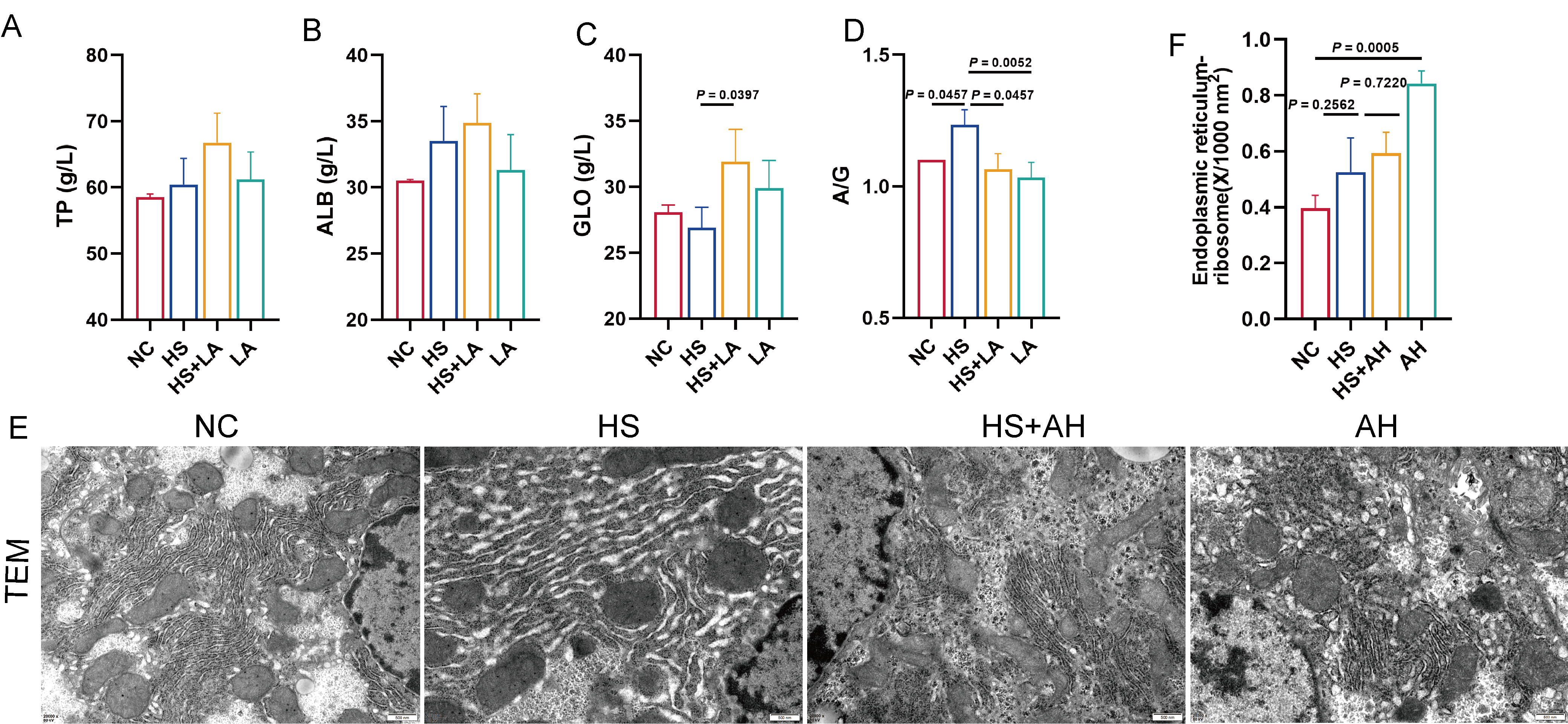

Supplement: Supplementary file 3 — Supplementary Material 3: Fig S2. AH promoted protein processing. A-D, Serum ALB, GLO and A/G were detected by a Biochemical analyzer. E, Representative images of the ultrastructure of liver cells. Scale bar, 500 nm. F, Endoplasmic reticulum-ribosome(X/1000 nm2) was counted by Image-J and CAD. Data are presented by the mean of biological replicates ± s.d. One-way analysis of variance was used for P-values. [file 44154_2024_178_MOESM3_ESM.tif]

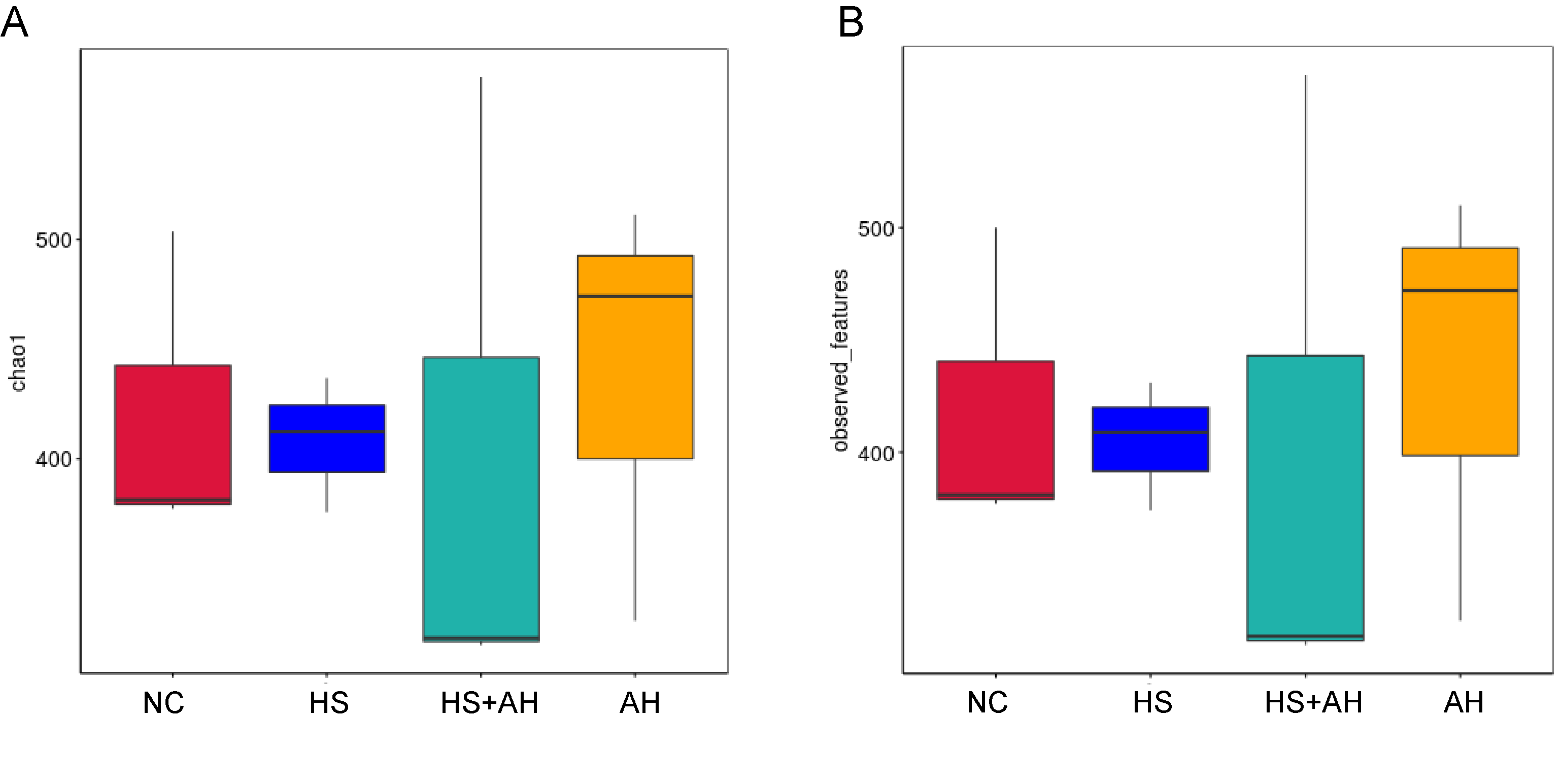

Supplement: Supplementary file 4 — Supplementary Material 4: Fig S3. The α-diversity including Chao1, observed features and dominance. A, chao1 index of each treatment group. B, observed_teatures index of each treatment group. [file 44154_2024_178_MOESM4_ESM.tif]
